# Hospital mortality of blunt abdominal aortic injury (BAAI): a systematic review and meta-analysis

**DOI:** 10.1186/s13017-023-00492-w

**Published:** 2023-03-29

**Authors:** Mingxuan Li, Yu Yan, Chaonan Wang, Haixia Tu

**Affiliations:** 1 Department of Vascular Surgery, Beijing Fengtai You’anmen Hospital, 199 Youanmenwai St, Fengtai District, Beijing, China; 2 Department of Vascular Surgery, Peking Union Medical College Hospital, Chinese Academy of Medical Science and Peking Union Medical College, Beijing, China

**Keywords:** BAAI, Blunt trauma, Abdominal injury, Mortality, Trauma

## Abstract

**Background:**

Studies on the mortality of blunt abdominal aortic injury (BAAI) are rare and have yielded inconsistent results. In the present study, we aimed to quantitatively analyse the retrieved data to more accurately determine the hospital mortality of BAAI.

**Methods:**

The Excerpta Medica Database, PubMed, Web of Science and Cochrane Library databases were searched to identify relevant publications without date restrictions. The overall hospital mortality (OHM) of BAAI patients was set as the primary outcome measure. English publications with data that met the selection criteria were included. The quality of all included studies was assessed by the Joanna Briggs Institute checklist and the American Agency for Health Care Quality and Research’s cross-sectional study quality evaluation items. After data extraction, a meta-analysis of the Freeman–Tukey double arcsine transformation of data was performed using the Metaprop command in Stata 16 software. Heterogeneity was assessed and reported as a percentage using the *I*^2^ index value and as a *P* value using the Cochrane Q test. Various methods were used to determine the sources of heterogeneity and to analyse the sensitivity of the computation model.

**Results:**

Of the 2147 references screened, 5 studies that involved 1593 patients met the selection criteria and were included. There were no low-quality references after assessment. One study that only included 16 juvenile BAAI patients was excluded from the meta-analysis of the primary outcome measure due to high heterogeneity. Due to the low heterogeneity (*I*^2^ = 47.6%, *P* = 0.126 for Q test) that was observed after using the random effects model, the fixed model was subsequently used to pool the effect sizes of the remaining four studies, thus yielding an OHM of 28.8% [95% confidence interval (CI) 26.5–31.1%]. The stability of the model was verified by sensitivity analysis, and Egger’s test (*P* = 0.339) indicated a low level of publication bias. In addition, we also performed meta-analyses and obtained a pooled hospital mortality of operation (13.5%, 95% CI 8.0–20.0%), a pooled hospital mortality of non-operation (28.4%, 95% CI 25.9–31.0%), and a pooled rate of aortic rupture (12.2%, 95% CI 7.0–18.5%) of BAAI.

**Conclusions:**

The present study indicated that BAAI has an OHM of 28.8%, indicating that this disease deserves more attention and research.

**Supplementary Information:**

The online version contains supplementary material available at 10.1186/s13017-023-00492-w.

## Introduction

Blunt abdominal aortic injury (BAAI) is caused by injury to the aorta related to blunt biomechanical direct and indirect forces incurred on the abdominal aorta (AA), which is tethered between the spinal column and the peritoneum and abdominal viscera [1]. According to previous reports, BAAI accounts for only 4–6% of all aortic injuries [2] and fewer than 1% of all blunt injuries [3, 4]. Additionally, its prevalence is fivefold lower than that of blunt thoracic aortic injury (BTAI) [5, 6]. Due to its rarity, there is a lack of studies on BAAI, with most of them being case reports. Thus, there is a limited understanding of the epidemiology, diagnostics, therapeutics, and prognostics of BAAI.

BAAI is fatal. The survival rate following aortic trauma induced by road traffic accidents has been reported to be less than 10% [7]. In addition, the prehospital mortality in emergency patients with blunt aortic injury (BAI) in the 1990s was reported to be close to 80% [8, 9]. Although there are no specific data, a large proportion of BAAI patients do not reach the hospital alive after experiencing trauma. Vascular surgeons focus on improving the hospital mortality of BAAI. Understanding more characteristics of BAAI, including its hospital mortality, will allow evaluation of prognoses and development of appropriate treatment modalities as soon as possible after such patients are sent to the hospital. There have been many controlled studies [10–13] on the mortality of BTAI overall and in various subgroups. A guideline [9] on the evaluation and management of BAI was published in 2015, but previous reports of the hospital mortality of BAAI vary widely. Roth et al. [14] reported a 24% hospital mortality in 1997, while Deree et al. [15] reported a value of 92% (probably including deaths on arrival) in 2007. After an extensive search, we did not identify any quantitative pooled analysis on the hospital mortality of BAAI. Therefore, we conducted a systematic review and meta-analysis on this issue.

## Methods

This review was registered with PROSPERO (CRD42022347794, https://www.crd.york.ac.uk/PROSPERO/display_record.php?RecordID=347794) and was performed in accordance with the Preferred Reporting Items for Systematic Reviews and Meta-Analyses (PRISMA) framework [16]. The checklist for the present study is shown in Additional file 1. Because all data analyses were based on original studies, no additional ethical approvals or consent forms for the participants were needed.

### Search and selection

The Excerpta Medica Database (Embase), PubMed, Web of Science (WOS) and Cochrane Library databases were systematically searched (date of search: July 21, 2022). We searched for all relevant articles without date restrictions using the following Medical Subject Headings (MeSH): “abdominal”, “aortic”, “injury” and all possible synonyms. All search terms are included in Additional file 2.

All references were imported into Endnote X9 for to remove duplicates and screen the study information. The full texts of all available articles that passed the preliminary screening were then downloaded and read to identify those that could be included in the current meta-analysis. Additionally, the bibliographies and citations of the included articles were screened to identify potentially eligible articles.

If an abdominal aortic injury was not caused by penetrating external forces or direct damage to the aortic wall, it was considered a BAAI. We also defined overall hospital mortality (OHM) as the overall mortality rate during hospitalization of those who arrived at the medical institution alive. In the present study, the OHM of BAAI was set as the primary outcome measure, while hospital mortality of operation (HMO, which included open surgery and endovascular therapy), hospital mortality of non-operation (HMNO), and rates of some diagnostic variables on disease severity were set as secondary outcome measures. Regardless of the type of study and year of publication, all retrieved original articles were included whenever they reported the OHM in BAAI patients or reported data that could be used to calculate OHM. The exclusion criteria were as follows: (1) articles not published in English; (2) the study subjects were animals; (3) studies with a sample size of less than 10; (4) studies whose data were completely contained within other studies already included; and (5) studies that were retrospective reviews of data reported in other studies, such as systematic reviews. Two authors (YY and CW) independently performed the title abstract screen and full-text review based on the above selection criteria. Any discrepancies were resolved by consensus or in consultation with ML.

### Data extraction

After identifying the included articles, all available data, including that on publication, sample demography and epidemiology, were extracted. If some data on outcome measures in a study were not reported directly, we calculated the outcome from the data given (e.g. the number of deaths equalled the number of sufferers multiplied by the mortality rate). Data extraction was performed by a pair of independent authors (ML and YY) manually rather than automatically. Any queries and discrepancies were resolved through further discussion to reach a consensus.

### Quality assessment

All the included articles were assessed by the Joanna Briggs Institute (JBI) checklist [17] and the American Agency for Health Care Quality and Research’s (AHQR) cross-sectional study quality evaluation items [18].

The JBI’s quality assessment tool for prevalence research includes nine items that evaluate the overall quality of prevalence research in terms of sampling methods, research objects, data collection, and analysis methods; the item is scored 1 point if the answer is “yes” and scored 0 points if the answer is “no”, “not clear” or “not applicable”. The AHQR’s cross-sectional study quality evaluation items contain eleven domains; “yes” is scored 1 point, and “no” or “not clear” is scored 0 points.

Quality assessment was performed by a pair of independent authors (ML and CW). Any queries and discrepancies were resolved through further discussion to reach a consensus. All included articles were classified as having “low” (0–3 points), “medium” (4–7 points) or “high” (8–11 points) methodological quality. For each article, the lower quality class between the two assessment systems was adopted.

### Statistical analysis

Stata (Stata Corp., College Station, TX, USA) version 16.0 was used for all statistical analyses. Meta-analyses of all outcome measures were performed using the Metaprop command [19] of the Freeman–Tukey (F-T) double arcsine transformation of data [20] to derive the pooled effect sizes (ESs) and 95% CIs. The fixed effects model or random effects model was used for the analyses, depending on the assessment of statistical heterogeneity [21]. In addition to textual description, the pooled analysis results of outcome measures of interest are presented as forest plots.

### Heterogeneity assessment and sensitivity analysis

In the present study, only models with low heterogeneity, as suggested by assessment, and with no fewer than three included studies were adopted. The heterogeneity across the studies was assessed and reported as a percentage using the *I*^2^ index value [22] and as a *P* value using the Cochrane Q test [23]. The analysis was performed using a random effects model first. If the *I*^2^ statistic was ≥ 50% or the *P* value was ≤ 0.10, the heterogeneity between the studies was high; otherwise, the heterogeneity was considered low. The random effects model was used to conduct a meta-analysis of the primary outcome measure. Any study causing high heterogeneity was removed from the model, and the differences between the OHM reported in the removed studies and those reported by the remaining studies were analysed by Fisher’s exact test [24]. *P* < 0.05 was considered statistically significant). When the pooled ES of the remaining studies suggested low heterogeneity, the fixed effects model was adopted. The initial plan was that a meta-regression analysis or a subgroup analysis would be performed to explore sources of heterogeneity if the extracted data could provide sufficient information.

To analyse the sensitivity of the resulting model, calculations were performed by changing the model type and omitting the included studies one by one.

### Publication bias assessment

We assessed the publication bias of the included studies using Egger’s test [25]. *P* < 0.05 indicated a significant difference. The initial plan was that if the number of studies included in the final model reached more than five, a funnel plot was generated to assess publication bias [26].

## Results

### Characteristics of studies and patients

**Fig. 1:**
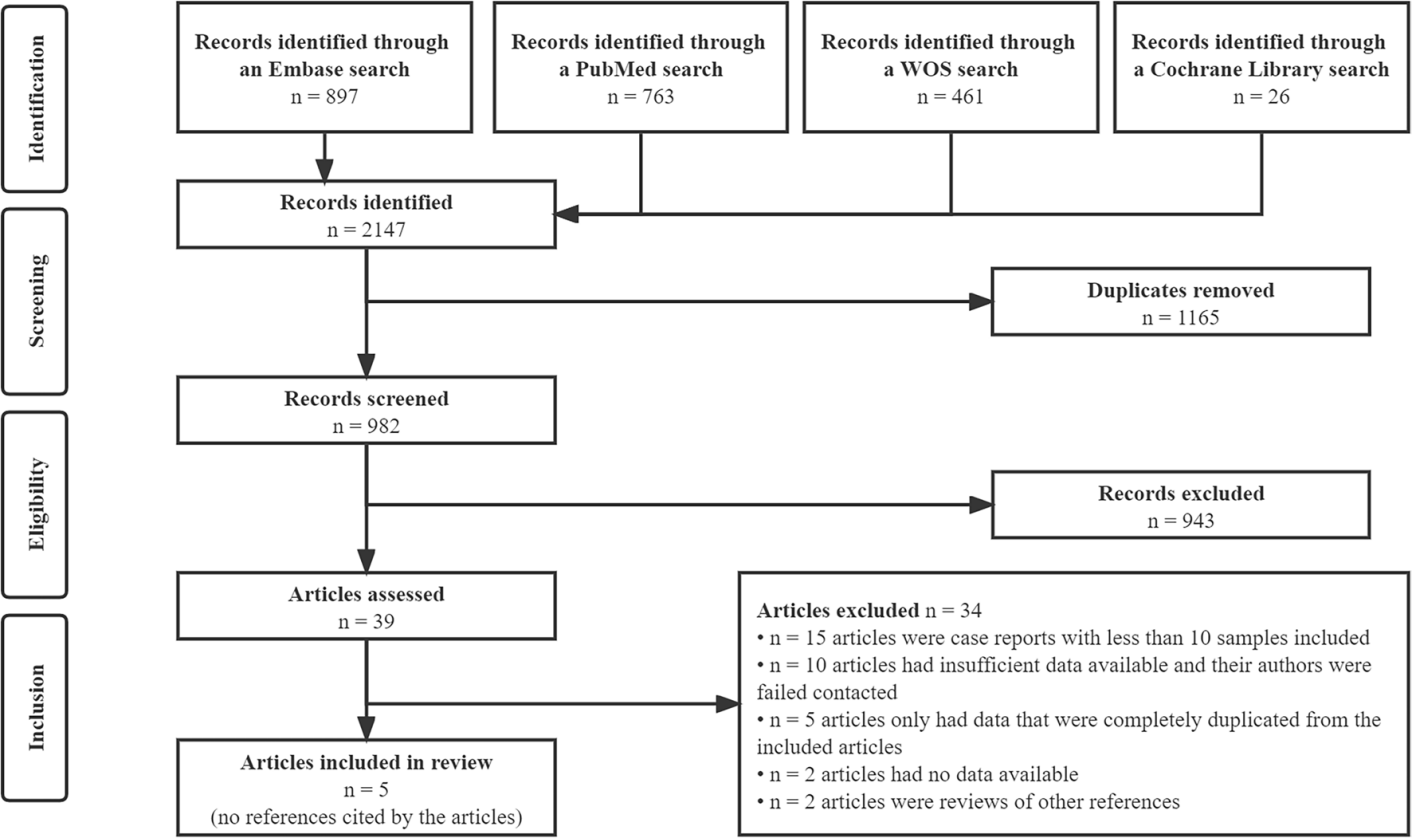
PRISMA flowchart of study selection

We initially retrieved 2147 articles from the 4 academic databases, of which 982 articles were evaluated after removing duplicates. In total, 39 articles were retained after screening the titles and abstracts. After reviewing the full texts, five articles [27–30] that met the selection criteria were included in the present study. None of the references cited by the screened articles were included. The PRISMA flowchart of study selection is shown in Fig. 1.

The 5 included studies had sample sizes ranging from 16 to 1012. There were 2 cross-sectional studies (single centre) and 3 case–control studies (multicentre), and all studies were retrospective. The extracted data spanned 1996–2019, and all studies were conducted in the United States of America (USA). A total of 1593 BAAI patients were included. Most of the patients were male, and the youngest age was 4 years old. The quality assessment showed that two articles were of high quality and three articles were of medium quality. The characteristics of the included studies and patients are summarized in Table 1.

**Table 1:** Characteristics of the 5 studies that met the selection criteria and the extracted data

First author (year)	Type of study	Score (JBI/AHRQ)	Source of data	Year criteria	Age criteria	Sample size	Prevalence of BAAI^c^ (‰)	Male (%)	Age and ISS (median or mean)	Injury in zone III^d^ (%)	Aortic rupture (%)	Operation^e^ (%)	OHM (%)	HMO (%)	HMNO (%)
Kim et al. [28]	Cross-section	6/9	SC^a^	2008–2019	< 18	16	N/A	50.0	Age 9, ISS 34	100	12.5	56.3	0	0	0
de Mestral et al. [4]	Case–control	7/8	NTDB	2007–2009	≥ 16	436	1.7	67.4	Age 46, ISS 35	N/A	N/A	9.6	29.4	11.9	31.2
Shalhub et al. [29]	Case–control	8/9	WTA	1996–2011	Unlimited	113	0.3	67.3	Age 38, ISS 34	66.4	31.9	61.9	38.9	45.7	27.9
Charlton-Ouw et al. [30]	Cross-section	7/10	SC^b^	2000–2014	Unlimited	16	0.3	68.8	Age 47, ISS 34	68.8	6.2	43.8	31.2	28.6	33.3
Sheehan et al. [31]	Case–control	8/8	TQIP	2010–2016	≥ 18	1012	1.0	71.9	Age 45, ISS 33	N/A	N/A	9.5	28.0	17.7	29.0

*JBI* the Joanna Briggs Institute; *AHRQ* the Agency for Healthcare Research and Quality; *SC* single centre; *NTDB* the National Trauma Data Bank; *WTA* the Western Trauma Association; *TQIP* the Trauma Quality Improvement Program; *N/A* not applicable; *BAAI* blunt abdominal aortic injury; *ISS* injury severity score; *OHM* overall hospital mortality; *HMO* hospital mortality of operation; *HMNO* hospital mortality of non-operation

^a^Texas Southwestern Medical Center

^b^Texas Medical Center

^c^Proportion of BAAI patients in those with blunt trauma

^d^zone III is the infrarenal aortic region

^e^Both open surgery and percutaneous intervention were included

### OHM of BAAI

**Fig. 2:**
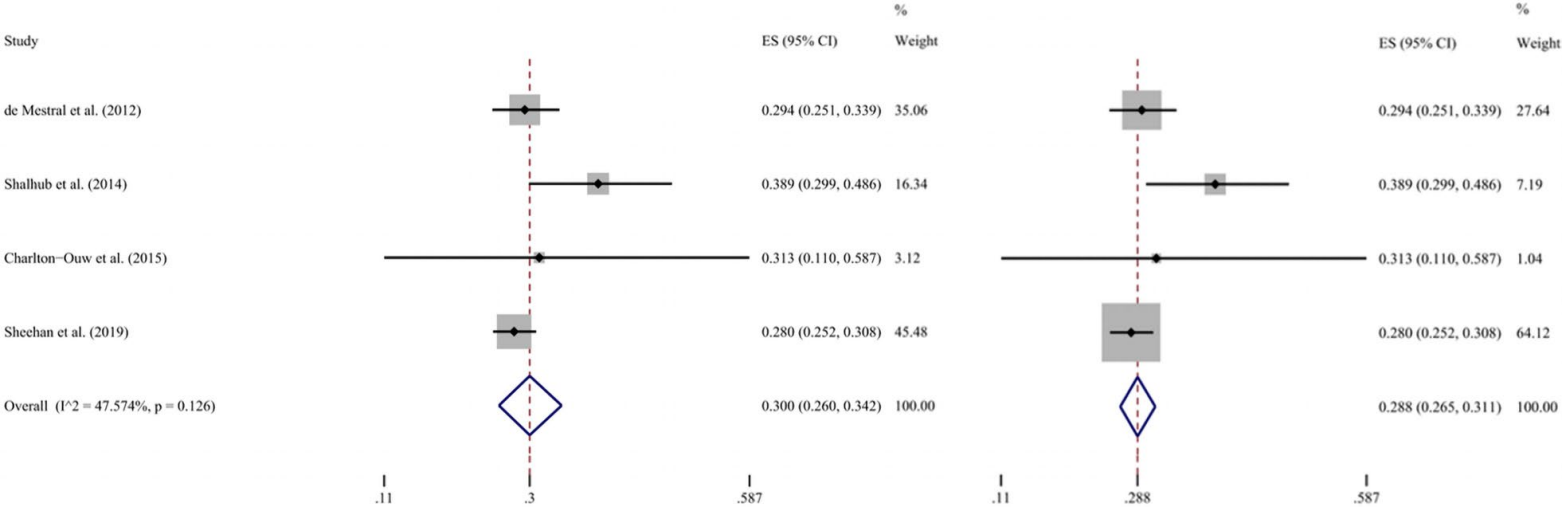
Forest plot of the overall hospital mortality in BAAI patients. The random effects model is shown left, and the fixed effects model right. *ES* effect size; *CI* confidence interval

After running the Metaprop command (metaprop event n, random ftt) in Stata 16, the results showed a high degree of heterogeneity (*I*^2^ = 78.8%, *P* = 0.001 for Q test). One study [27], which reported only 16 cases of BAAI and had a mortality rate of 0%, was removed due to potential severe heterogeneity. The remaining four studies reported OHMs ranging from 28.0 to 38.9%, and the random effects model indicates that the pooled ES was 30.1% (95% CI 26.3–33.9%, *I*^2^ = 47.6%, *P* = 0.126 for Q test). According to the established rules, the above ES was recalculated using a fixed effects model, and the pooled ES was 28.8% (95% CI 26.5–31.1%), which was adopted (Fig. 2).

### Heterogeneity and sensitivity

Fisher's exact test showed significant differences between the OHM reported by the excluded study and the OHMs reported by the other four studies (*P* values were 0.008, 0.001, 0.043, and 0.009). An assessment of the pooled ES of the remaining four studies using a random effects model yielded an *I*^2^ value of 47.6% and a *P* value of 0.126 for the Q test, which suggested low heterogeneity. Due to the small number of included studies and the paucity of data for many independent variables (such as aortic lesion grade or location), subgroup analysis or meta-regression analysis was not performed.

**Fig. 3:**
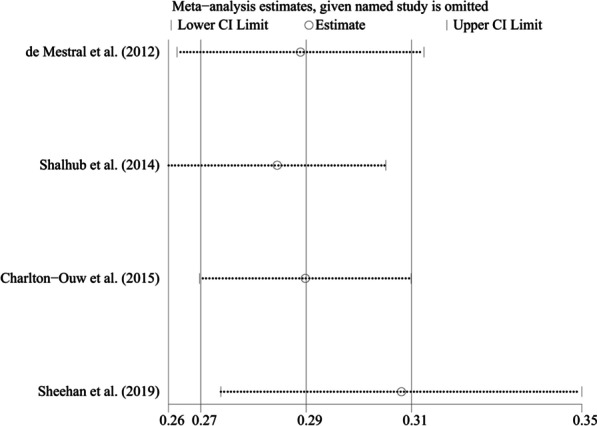
Sensitivity analysis chart for the meta-analysis of the overall hospital mortality using fixed effects model

The ES derived from the fixed effects model was similar to that derived from another model (28.8% vs. 30.0%, respectively). In addition, the four articles were omitted from the final model one at a time to analyse the sensitivity of the model, which yielded a satisfactory result (Fig. 3). Thus, these findings verified the low sensitivity of the final model for the OHM of BAAI.

### Publication bias

No funnel plot was drawn due to the small number of included studies. Egger’s test yielded a *P* value of 0.339, indicating low publication bias.

### Secondary outcome measures

**Fig. 4:**
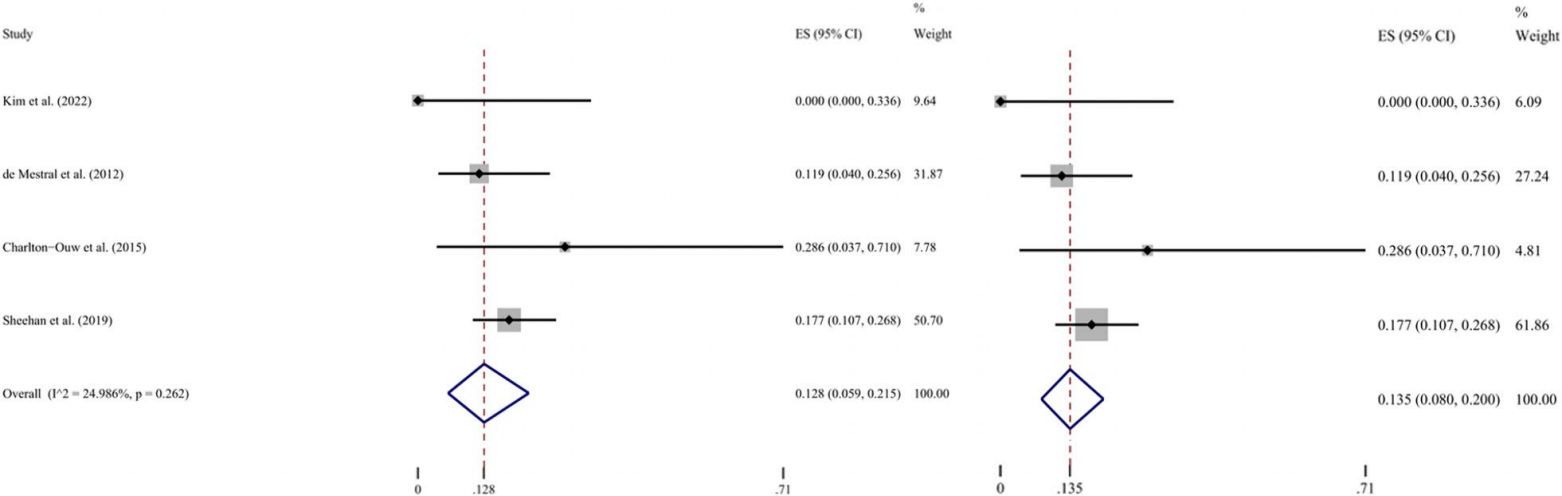
Forest plot of the hospital mortality of operation in BAAI patients. The random effects model is shown left, and the fixed effects model right. *ES* effect size; *CI* confidence interval

**Fig. 5:**
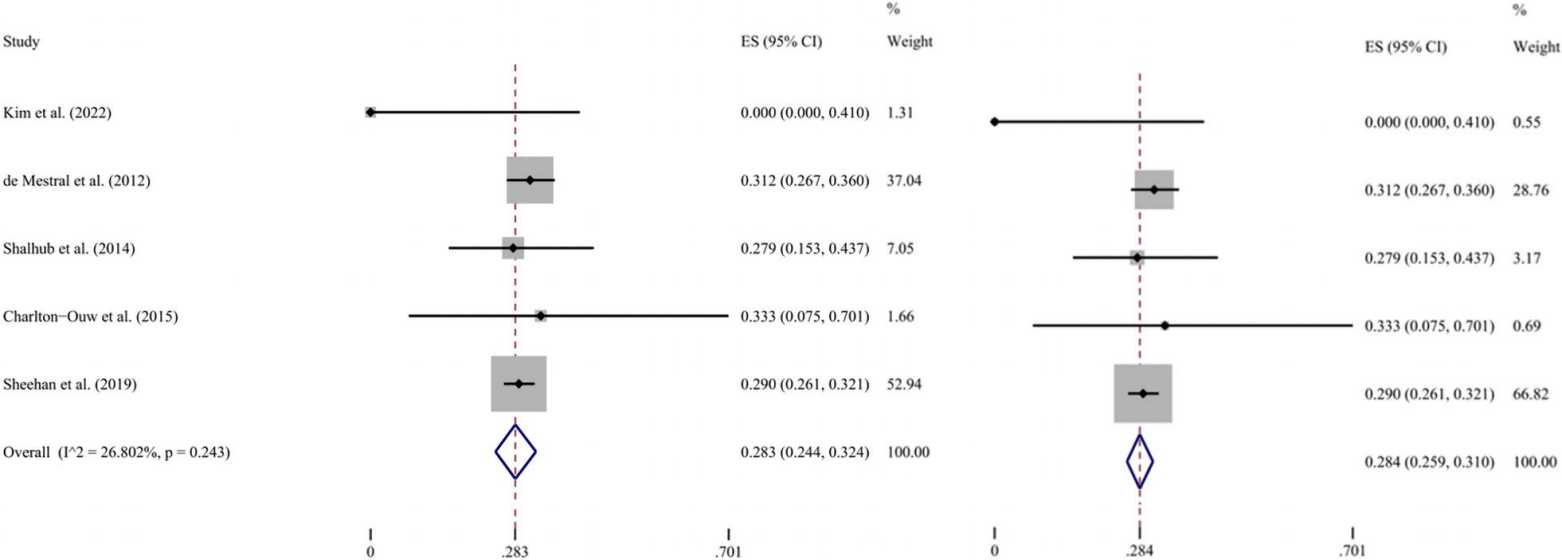
Forest plot of the hospital mortality of non-operation in BAAI patients. The random effects model is shown left, and the fixed effects model right. *ES* effect size; *CI* confidence interval

**Fig. 6:**
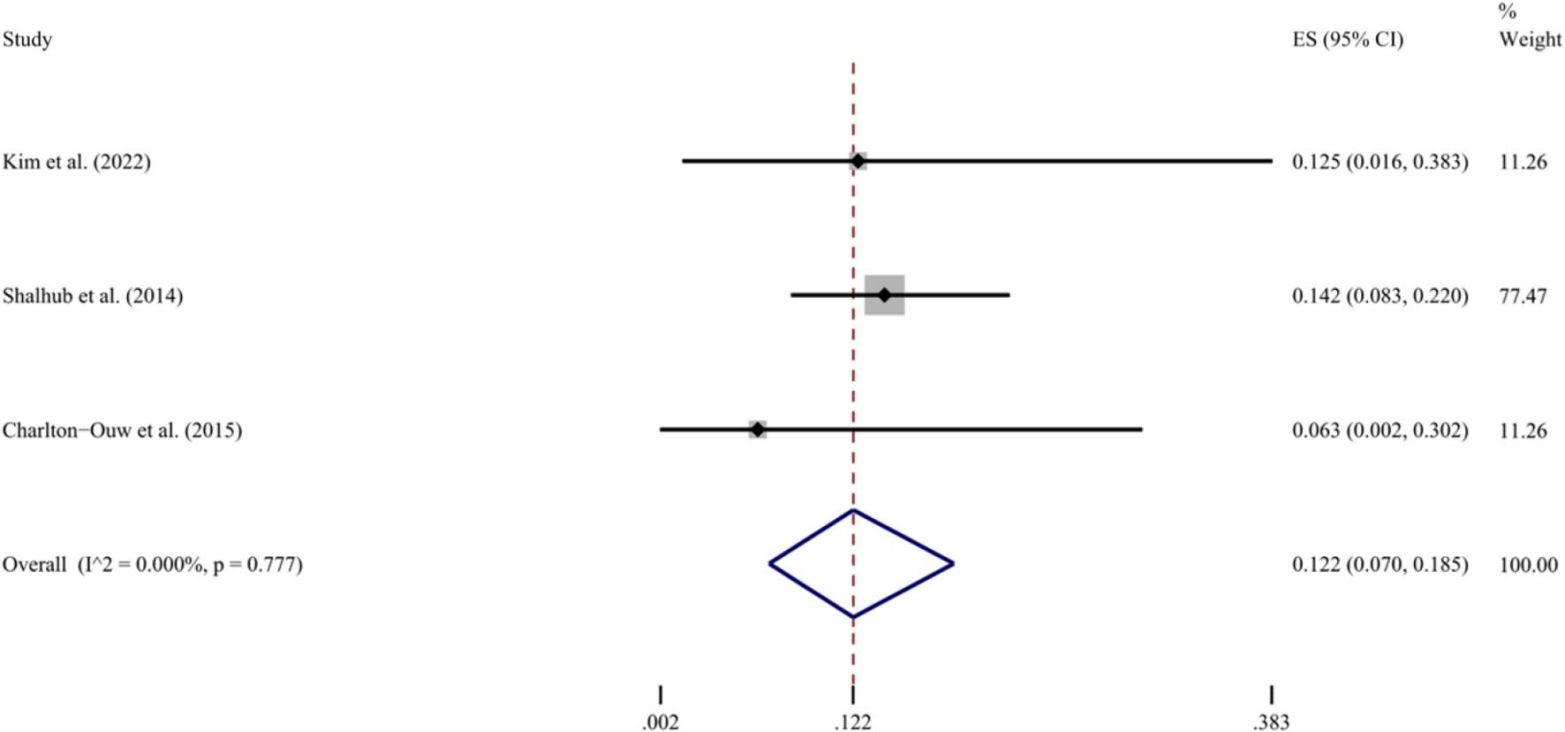
Forest plot of the rate of aortic rupture by BAAI (random effects model). *ES* effect size; *CI* confidence interval

The pooled analysis results of the five included studies regarding the HMO showed high heterogeneity (*I*^2^ = 88.4%, *P* < 0.001 for Q test). When the study that reported an ultrahigh mortality of 45.7% [28] was excluded, low heterogeneity (*I*^2^ = 25.0%, *P* = 0.262 for Q test) was obtained, with a resulting pooled HMO of 13.5% (95% CI 8.0–20.0%) (Fig. 4). Using the same process, a pooled HMNO of 28.4% (95% CI 25.9–31.0%) was obtained with no excluded study and low heterogeneity (*I*^2^ = 26.8%, *P* = 0.243 for Q test) (Fig. 5). Additionally, the rates of infrarenal aortic injury and aortic rupture were extracted and pooled for analysis based on the available data after extraction. The proportion of infrarenal aortic injury in those of all AA locations was reported by three studies, ranging from 66.4 to 100% (Table 1). After analysis, the results showed high heterogeneity (*I*^2^ = 88.1%, *P* = 0.001 for Q test), which did not meet the requirements of the present study. Due to the small sample size, the source of heterogeneity was not identified through meta-regression or subgroup analysis. The same three studies reported aortic rupture rates ranging from 6.2 to 12.5% (Table 1). Analysis using the random effects model indicated low heterogeneity (*I*^2^ = 0%, *P* = 0.777 for Q test), and this result was the same as that obtained using the fixed effects model, i.e. the pooled rate was 12.2% (95% CI 7.0–18.5%) (Fig. 6).

## Discussion

### Significance of this study

BTAI is more common than BAAI [5, 6] and had thus been more widely studied [9–13]. BTAI has even been considered equivalent to BAI in a broad sense [9]. In 2009, Demetriades et al. [12] reported that the overall mortality of BTAI was only 12.4%, while Fox et al. [9] reported that the mortality in BTAI patients undergoing thoracic endovascular aortic repair was as low as 9% in a large sample meta-analysis in 2015. The reason for the relatively low mortality may be due to the accumulation of substantial clinical experience. In contrast, BAAI has a significantly higher mortality. In the present study, we found that the reported lower hospital mortality still reached 28.0% [30]. Because the mortality is significantly higher and the anatomical features and treatment modalities are quite different, BAAI is considered a completely different disease from BTAI. In contrast to BTAI, there are only a few studies on BAAI, and most of them are case reports. Because there were no previous meta-analyses focusing on BAAI alone, the present study was performed to evaluate the prognosis of BAAI patients more accurately, thus enabling BAAI patients to receive more timely and accurate treatments.

### Sources of data

In addition to the five included studies, four studies containing potentially adequate patients attracted our attention. Of them, two studies [31, 32] did not report the number of BAAI patients, one study [33] included 20 patients without reported mortality, and one study [15] reported a 92% mortality, including prehospital death. Unfortunately, we have not been able to obtain the relevant data of these studies.

Five studies [1, 5, 34–36] were excluded due to complete duplication of data. However, three [4, 28, 30] of the included studies may have some degree of duplication because the centres may have been duplicated across these multicentre studies. Considering that the data duplication was not serious according to the comparisons of their inclusion criteria (year, age, etc.), these three studies were included in the present study, which may have increased the error of the present study to some extent.

In addition, the five included studies were all from the USA. Except for the excluded article that exclusively included juveniles and had a sample size of only 16 [27], 2 of the remaining 4 exclusively included adults [4, 30]. These factors may reduce the fitting effect of the results of the present study for populations overall.

### Analyses and outcomes

In the process of extracting data, we determined all the variables with data that could be displayed or analysed. In addition to the primary outcome measure (i.e. OHM), the HMO and HMNO of BAAI in the studies were also calculated [4] or directly extracted, and they were considered the secondary outcome measures. Due to the lack of other available data on BAAI patients in these articles, however, it was difficult to perform other quantitative analyses, such as comparison of mortalities from various treatment modalities and screening of predictors for death.

Because this study was a single-arm meta-analysis involving no control groups, we considered all included studies for quality assessment as simple observational studies without intervention. The result that no low-quality studies were found was satisfactory.

Due to a higher statistical performance and applicability to samples with a very low value [37], we performed the F-T double arcsine transformation of data before the meta-analysis, which reduced the risk of blind exclusion of the above described studies. Unfortunately, the study reporting an OHM of 0% was still excluded due to high heterogeneity (*I*^2^ = 78.8%, *P* = 0.001 for Q test). Of note, this was a single-centre study with a small sample size that only enrolled paediatric individuals < 18 years of age.

We were unable to determine the impact of the excluded study on each of the three variables, namely year of cases, gender, and injury severity score [38]. To explore the reasons for the extremely low mortality in this study, we investigated the factors or variables that may affect the risk of death in BAAI patients. Unfortunately, after an extensive search, it appears that only one study [30] has performed this work, suggesting that in adult BAAI patients, increased age is a risk factor for death. However, it is not known whether this conclusion applies to paediatric patients. Due to the limited data, we were unable to evaluate the differences in other factors affecting mortality between the excluded study and the other four studies [30].

It is worth noting that the study [30] that explored predictors of death in BAAI did not include the location and grade of aortic lesions, which are two important variables. Shalhub et al. [1] reported that the AA is divided into three zones [zone I, above the superior mesenteric artery (SMA); zone II, from the SMA to renal artery; and zone III, infrarenal] to guide the operation for BAAI, which is a widely recognized method [27–29, 39, 40]. Although this study was excluded from the present meta-analysis of OHM because its data were completely duplicated with one [28] of the included studies, it reported mortality rates of BAAI by aortic lesion location, i.e. 60% in zone I, 100% in zone II, and 15% in zone III. In combination with the opinions of Shalhub et al. [1], we consider that the reason for the significantly lower mortality in zone III is that compared to those in the other two zones, the aortic injuries in this zone are less complicated with the injuries of other organs or aortic branches, and they are easier to expose by open surgery or to repair endovascularly. Of note, all the locations of aortic lesions in patients in the excluded study [27] were zone III, whereas the other two available studies reported the proportion of this lesion location to be 66.4% [28] and 68.8% [29], respectively. Although the pooled analysis was not completed due to high heterogeneity to obtain a specific value, we found that zone III injuries accounted for the majority, providing some confidence to clinicians and patients. Moreover, the grade of BAAI lesion severity should be defined. At present, there is no consensus in this regard specifically for BAAI. The assessment methods of all previous studies were the same as those of BTAI, and it remains unknown whether they apply to BAAI. Azizzadeh et al. [41] classified the aortic lesions of BTAI patients into four grades as follows: internal tear, intramural haematoma, pseudoaneurysm, and rupture. Rabin et al. [42] utilized a different classification standard as follows: internal tear or intramural haematoma, small pseudoaneurysm (< 50% of the aortic circulation), large pseudoaneurysm (> 50% of the aortic circulation), and rupture or transection. The pooled analysis yielded an aortic rupture rate of 12.2%, thus indicating the mortality risk of BAAI. Due to the paucity of studies and data available, we were unable to determine the relationships between the location and severity of aortic lesions and the risk of mortality in BAAI patients by quantitative calculations. However, we believe that patients who have aortic lesions that are more easily repaired or of lesser severity will have a lower risk of mortality.

In the excluded study [27], the mechanisms of injury for all patients were all seat belt-related motor vehicle accidents. Although the rate of seat belt-related mechanisms could not be extracted directly from the three included studies [4, 28, 30] that reported relevant data due to nonuniformity in classification standards and missing data, the rate is expected to greatly differ from 100%. It is unknown whether this difference is one of the reasons for the large difference in OHM. The ambiguity in the classification standard for the mechanisms of injury in BAAI represents insufficient knowledge among investigators in this regard. In 1962, BAAI and simultaneous lumbar spine fracture were initially described as “seat belt syndrome” [1]. BAAIs with seat belt injury mechanisms are not uncommon in case reports [43–46]. Additional studies are required to understand whether there are essential differences among the injury mechanisms of seat belts, those of motor vehicle accidents without seat belts, and even those of nonmotor vehicle-induced trauma to cause different death risks from BAAI.

In summary, a lower mean age, high proportions of seat belt-related injury mechanisms and injuries in zone III may contribute to the unusually low mortality (as low as zero) in the excluded study [27]. However, if the sample size increases, the OHM may be valuable.

Of the four included studies, two studies included only adults (≥ 16 years old [4] and ≥ 18 years old [30]) without explanations. In addition, one study [29] had a sample size of only 16 cases, which was small compared to the other studies, and the differences between the data available for extraction were not significant. Therefore, the differences in age and the small sample size may explain the heterogeneity among the studies.

After the heterogeneity among the included studies was demonstrated to be low (*I*^2^ = 47.6%, *P* = 0.126 for Q test), we adopted the fixed effects model to determine a true pooled ES [47]. The OHM of BAAI was found to be 28.8% (95% CI 26.5–31.1%). This rate of nearly 30% of BAAI varied from the 10% rate of BTAI, confirming our hypothesis that they are two different diseases.

This result was consistent with that obtained by the random effects model (30.1%, 95% CI 26.3–33.9%), and no ES was found to be outside the previous 95% CI after omitting studies one by one, which demonstrated that the final model had good stability. The *P* value obtained by Egger’s test was 0.339, which was far higher than 0.05, suggesting that the model had a low level of publication bias.

In addition, through statistical analyses by the same method, we also obtained an HMO of 13.5% and an HMNO of 28.4%. Although the included studies were slightly different, the significantly lower hospital mortality (13.5%) suggests that the operation has great benefits for BAAI patients compared to simple observation. However, it is necessary to hierarchically consider which treatment modality is more beneficial for each BAAI patient. Because BAAI is a fatal disease and that operation is a good treatment choice, it is important to define which patients are at higher risk of death and need prompt operation. Thus, the predictors of hospital death in BAAI patients need to be identified, which will allow more rational treatment.

### Strengths and limitations

To the best of our knowledge, the present study is the first meta-analysis on the mortality of BAAI. This study provided insight into the mortality of BAAI, a rare but fatal disease, through an extensive search and scientific analysis. However, this study had several limitations. First, there may be a small degree of duplication in the samples of the included multicentre studies, which may increase the statistical error. Second, the number of studies included in the analysis was small, which may reduce the statistical power.

## Conclusions

The present meta-analysis estimated the hospital mortality of BAAI patients to be 28.8% (95% CI 26.5–31.1%) overall, 13.5% (95% CI 8.0–20.0%) with operation, and 28.4% (95% CI 25.9%–31.0%) without operation. Further exploration of the predictors of death in BAAI is still needed.

## Supplementary Information

**Additional file 1**. PRISMA 2020 checklist.

**Additional file2**. Search terms forliteratures.

## Data Availability

The datasets used and/or analysed during the current study are available from the corresponding author on reasonable request.

## Abbreviations

AA: Abdominal aorta
AHQR: Agency for Healthcare Quality and Research
BAAI: Blunt abdominal aortic injury
BAI: Blunt aortic injury
CI: Confidence interval
Embase: Excerpta Medica Database
ES: Effect size
F-T: Freeman–Tukey
HMNO: Hospital mortality of non-operation
HMO: Hospital mortality of operation
JBI: Joanna Briggs Institute
MeSH: Medical subject headings
OHM: Overall hospital mortality
SMA: Superior mesenteric artery
USA: United States of America
WOS: Web of Science
