# Supplementary material for: Hospital mortality of blunt abdominal aortic injury (BAAI): a systematic review and meta-analysis
Source: World J Emerg Surg. 2023 Mar 29;18:26. doi: 10.1186/s13017-023-00492-w (PMC10061949; doi:10.1186/s13017-023-00492-w)
Supplement: Supplementary file 2 — Additional file2. Search terms forliteratures. [file 13017_2023_492_MOESM2_ESM.docx]

**Search terms used in the literature research on different platforms:**

**Embase**

(injuries:ti OR wounds:ti OR injury:ti OR wound:ti OR trauma:ti OR traumas:ti OR traumatic:ti) AND (aorta:ti OR aortas:ti OR aortic:ti) AND (abdomens:ti,ab,kw OR abdomen:ti,ab,kw OR abdominal:ti,ab,kw OR stomachs:ti,ab,kw OR stomach:ti,ab,kw OR belly:ti,ab,kw)

**PubMed**

("aorta"[Title] OR "aortas"[Title] OR "aortic"[Title]) AND ("injuries"[Title] OR "wounds"[Title] OR "injury"[Title] OR "wound"[Title] OR "trauma"[Title] OR "traumas"[Title] OR "traumatic"[Title]) AND ("abdomens"[Title/Abstract] OR "abdomen"[Title/Abstract] OR "abdominal"[Title/Abstract] OR "stomachs"[Title/Abstract] OR "stomach"[Title/Abstract] OR "belly"[Title/Abstract])

**WOS**

((TI=(injuries or wounds or injury or wound or trauma or traumas or traumatic)) AND TI=(aorta or aortas or aortic)) AND TI=(abdomens or abdomen or abdominal or stomachs or stomach or belly)

**Cochrane Library**

(injuries or wounds or injury or wound or trauma or traumas or traumatic):ti AND (aorta or aortas or aortic):ti AND (abdomens or abdomen or abdominal or stomachs or stomach or belly):ti,ab,kw
